# Immune checkpoint inhibitors for unresectable or metastatic pleomorphic dermal sarcomas

**DOI:** 10.3389/fonc.2022.975342

**Published:** 2022-11-17

**Authors:** Doris Helbig, Sebastian Klein

**Affiliations:** ^1^ Department of Dermatology, University Hospital Cologne, Cologne, Germany; ^2^ Department of Hematology and Stem Cell Transplantation, University Duisburg-Essen, University Hospital Essen, Essen, Germany

**Keywords:** Immune checkpoint inhibitor, PD-1, PD-L1, pleomorphic dermal sarcoma (PDS), unresectable, metastasized

## Abstract

Pleomorphic dermal sarcomas (PDS) are rare neoplasms of the skin that occur in UV-exposed sites in the elderly, but represent the most common cutaneous sarcomas. Although the majority of PDS can be surgically removed, local recurrences occur in up to 28%, usually occurring within the first two years after primary excision. Metastases are diagnosed in up to 20% of cases, mainly observed in the skin, lymph nodes and lungs, preferentially affecting patients with underlying hemato-oncologic diseases. Similar to other UV-induced tumors, PDS are inflammatory and immunogenic tumors (with a high number of CD4+/CD8+ tumor-infiltrating lymphocytes (TILs) and checkpoint molecule expression such as PD-L1, LAG-3, TIGIT) with a very high mutational burden. The most common genetic alterations include UV-induced *TP53* loss of function mutations, followed by alterations in the *CDKN2A/B* gene. Rarely, targetable genetic alterations can be detected. Compelling experimental data and clinical reports about PD-1/PD-L1-blocking antibodies in patients with PDS suggest its use as first line treatment in unresectable or metastatic tumor stages. However, individual („off-line”) patient management should be discussed in an interdisciplinary tumor board based on molecular genetic testing, mutational burden, PD-L1 expression, and evidence of tumor-infiltrating lymphocytes in addition to comorbities of the individual patient.

## Introduction

Pleomorphic dermal sarcomas (PDS) are rare neoplasms of the skin with a mesenchymal (fibroblastic) lineage differentiation, arising in UV-exposed locations, typically diagnosed in elderly male individuals (1–3). Although accurate incidence data do not exist, they represent the most common cutaneous sarcomas with increasing incidence due to demographic changes.

Given the similarities in clinic, histology as well as molecular genetics and epigenetics, atypical fibroxanthoma (AFX) and PDS are now considered a spectrum of one entity, but differ in terms of therapy and prognosis (4–10). Histomorphologically, AFX and PDS show similar features. The main difference is that AFX are confined to the dermis whereas PDS involves distinct portions of the subcutis and/or have necrotic tumor portions and/or perineural or lympho-vascular invasion. In AFX, the local recurrence rate after R0 resection is less than 5% (3, 11, 12). While the majority of PDS can be treated by curative excisions, local recurrences occur in up to 28% of patients. Metastases are observed in up to 20%, mainly in the skin, lymph nodes and lungs, preferentially affecting patients with underlying hemato-oncologic diseases (3, 10, 11, 13, 14).

In the last years, it could be shown that PDS are inflammatory and immunogenic tumors with a very high mutational burden. Experimental data and clinical reports indicate that PD-1/PD-L1-blocking antibodies are highly effective in patients with unresectable or metastatic PDS and suggest its use as first line treatment in these advanced tumor stages (2, 15–17).

## Genetic alterations

**Figure 1:**
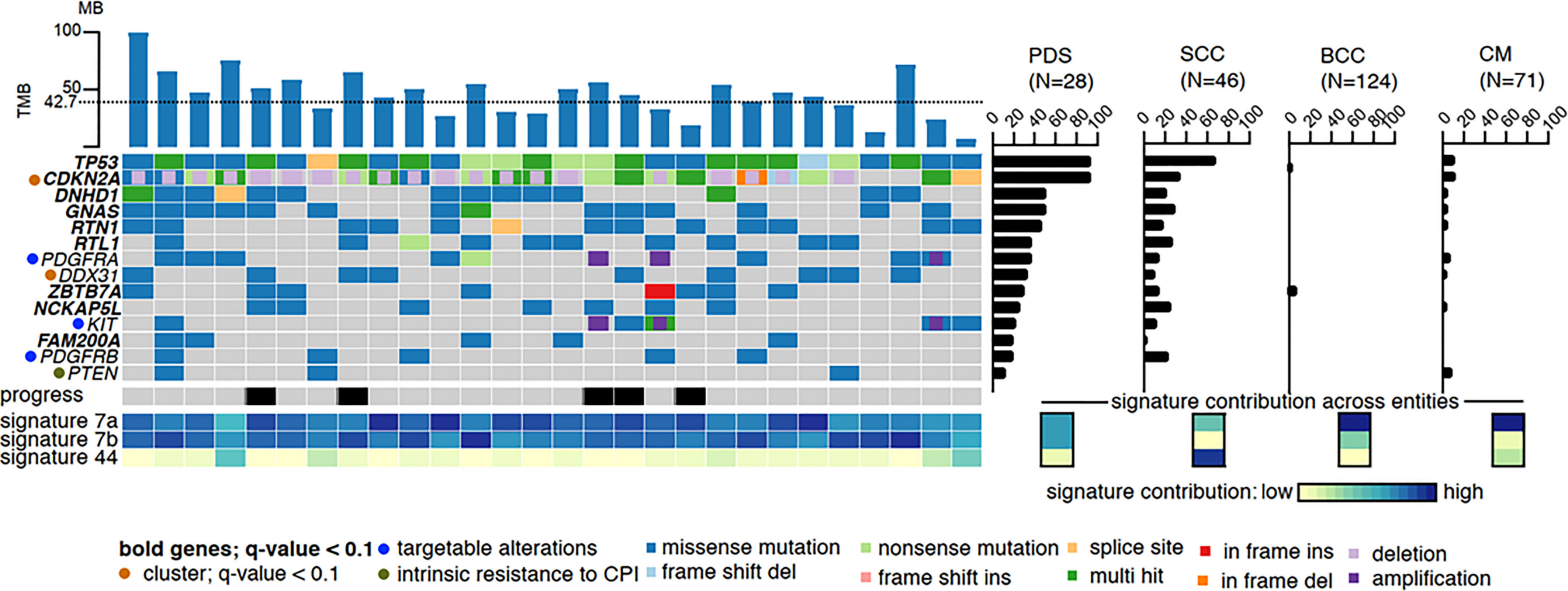
High mutational burden and most common mutations of PDS: The dotted line represents the average of variants per megabase. The mutational frequency of pleomorphic dermal sarcoma (PDS) is compared to cutaneous squamous cell carcinoma (SCC), basal cell carcinoma (BCC), and cutaneous melanoma (CM), where the bars indicate their relative prevalence (percentage, right panel). Cross entity comparison of mutational signatures shown below the right panel of mutational frequencies. Clinically, locally or systemically progressed tumors are indicated with a black bar (2).

Both, AFX and PDS have a very high mutational burden with UV signature, which is even higher than that of other UV-induced skin tumors such as cutaneous squamous cell carcinomas (cSCC) and malignant melanomas (2, 18–20). Based on very similar gene mutations, gene expressions, copy number variations as well as DNA methylation profiles, AFX and PDS are now accepted to represent a spectrum of the same tumor entity (5, 7–9). In PDS, it has been shown that the tumors exhibit the UV-induced mutation signatures 7a and 7b in almost equal proportions. In other UV-induced tumors such as cSCC, basal cell carcinoma, and melanoma, signature 7a is typically detected, whereas signature 7b is rarely detected. Signature 44, which has been associated with defective DNA mismatch repair (MMR), has been detected in a small number of our investigated PDS (3 of 28); however, is much more common in cSCC (**Figure 1**) (2).

Gene mutation and gene expression analyses revealed that AFX/PDS have the highest similarity to cSCC. The most frequent genetic alterations are *TP53* loss of function mutations which can be detected in all PDS, followed by genetic alterations in the *CDKN2A/B* gene (*CDKN2A/B* mutations in 68%, deletions in 71%, and both in 46%, while 7% showed even a biallelic loss.) (**Figure 1**) (2). Other common mutations include *DNHD1*, *GNAS*, *RTN1*, *RTL1*, *ZBTB7A*, *NCKAP5L*, *FAM200A*, *NOTCH1/2*, *FAT1*, and *TERT* promoter mutations (2, 5, 6, 8, 20, 21). In contrast, cSCC, basal cell carcinomas and malignant melanomas show a significantly lower mutation frequency of these frequently mutated genes (2). In a small proportion of PDS, amplifications of *PDGFRA* leading to a PDGFRA expression on protein level and mutations within the kinase domain of *KIT* could be detected.

## Immunophenotyping

Immunohistochemical as well as mRNA expression analyses of the immune “microenvironment” have shown that the majority of PDS represent inflammatory and immunogenic tumors with a high number of CD8+ tumor-infiltrating lymphocytes (TILs) and expression of diverse checkpoint molecules such as PD-L1, TIGIT, LAG-3, and CTLA-4 (2, 15, 16).

When we classified a series of PDS tumors into immunologically hot and cold tumors (high versus low amounts of both CD4/CD8+ cells and high versus low PD-L1 expression), we did not detect a significant difference of tumor mutational burden (TMB). Nevertheless, the TMB is usually high in almost all PDS cases. Differential gene expression analysis between these immunologically hot and cold tumors revealed upregulation of TIGIT in the immunologically hot tumors (2).

In general, elevated levels of immune-related cytokines such as IL1A, IL2, as well as markers that were very recently linked to enhanced response of immunotherapy in malignant melanoma, including CD27, and CD40L have been detected in PDS tumors (22, 23). Moreover, the majority of PDS showed strong MHC-I expression and upregulated HLA class I molecules (*HLA-A, HLA-B, HLA-C* and *HLA-E*, corresponding in humans to the MHC class I) that are involved in tumor neoantigen presentation to tumor-specific CD8+ T lymphocytes leading to tumor cell apoptosis (24–28).

In CD8+high PDS cases (defined as cases with CD8 levels above median), genes such as *CD74*, *LYZ* and *HLA-B* were found to be differentially expressed while the remaining cases revealed enhanced levels of immunosuppressive cytokines including *CXCL14* (29). In addition, the majority of PDS was infiltrated by PD-L1-, PD-1- and LAG-3-expressing immune cells and showed strong MHC-I expression on tumor cells (15).

These results imply that PDS in general, but especially those with a lot of infiltrating CD8+ and/or PD-L1- and LAG-3-expressing TILs as well as MHC-I expression, induce an adequate anti-tumor immune response, which could be enhanced by immune checkpoint inhibitors. Only a small proportion of tumors appear to develop “immune escape” mechanisms, such as downregulation of MHC-I molecules (2, 15, 16).

## Treatment of localized stage PDS

Radical excision followed by histopathologic workup is usually performed with curative intent as initial treatment for PDS. An appropriate safety margin should be maintained, as the risk of local recurrence or metastasis can be reduced by wide local excision (3, 12, 13, 30, 31). If this is not possible, a microscopically controlled excision should be performed. Although there are no published data on the radiation sensitivity of AFX/PDS, radiation of the tumor area may be considered if complete tumor excision is not possible. The efficacy of adjuvant radiation with respect to the prognosis of completely excised PDS has not been conclusively established. In an evaluation of a few patients who had received adjuvant postradiation, a positive tendency (fewer local recurrences and/or metastases) of this postradiation could be elicited (3).

## Treatment of advanced stage PDS including immune checkpoint inhibition

In case of advanced stage PDS, therapy recommendations should be always discussed and issued in the context of an interdisciplinary tumor board because there is no proven standard therapy. Here, molecular genetic testing, mutational burden, PD-L1 expression, and evidence of tumor-infiltrating lymphocytes (TILs) should be incorporated into individual treatment recommendations.

Since PDS harbor a high mutational burden and mostly exhibit an inflamed, proimmunogenic tumor microenvironment, susceptibility to immune checkpoint inhibition by programmed cell death 1 (PD1)/programmed cell death ligand 1 (PD-L1) inhibitors, (e.g. pembrolizumab, nivolumab) or the anti–CTLA-4 antibody (ipilimumab), or a combination of these agents was presumed in reference to other highly mutated and immunogenic tumors including other skin tumors such as malignant melanoma and cSCC (18, 32, 33). In the meantime, the exceptionally high efficacy of the anti-PD-1 inhibitor pembrolizumab has been described in case reports or small case series (see **Table 1**) (2, 15–17). Until now, there are no case reports describing the use of other CPI in PDS patients. Nevertheless, larger clinical studies are needed to investigate tumor response to CPI in PDS patients.

**Table 1:** Clinical case reports of PDS patients receiving CPI.

Authors	Patient (M/F; age in years)	PDS location	Treatment	TMB	PD-L1 expression	CD8+ TILs	Treatment outcome	Immuno-suppression
Klein S. et al. (2, 16)	1. M;77	Recurrence on the forehead + multiple parietal cutaneous metastases	Pembrolizumab (2 mg/kg/3 weeks)	63.2/MB	Moderate infiltration of PD-L1+ TILs	Moderate	Complete remission after 8 cycles as well as sustained response over 4 years (discontinuation of CPI treatment after 21months)	No
	2. M; 88	Inoperable primary (temporal)	Pembrolizumab (200 mg/3 weeks) combined with local RT (70 Gy using 6 MeV electrons in a linear accelerator)	78.0/MB	Limited PD-L1 expression of tumor and TILs	Moderate	Complete remission after 4 months. Patient died shortly thereafter due to CLL progression	CLL
	3. F; 79	Inoperable primary on the cheek with infiltration of the mandibular bone	One single dose of Pembrolizumab, stopped due to autoimmune colitis (grade II)	NA	TPS=80%	NA	Tumor meltdown after single infusion	CLL
Klein O. et al. (17)	M; NA	2 cutaneous metastases on the scalp, parotid gland metastases, 2 lung metastases	Pembrolizumab	NA	NA	Heavy lymphocytic infiltration	Complete remission after 3 months, treatment stop after owing to a flare of pre-existing polymyalgia rheumatica	No

CPI, checkpoint inhibition; M/F, male/female; TILs, Tumor-infiltrating lymphocytes; TMB/MB, Tumor mutational burden/megabases; TPS, Tumor Proportion score; RT, radiotherapy; NA, Not applicable.

For PDS cases with low levels of CD8+ TILs, interventions to increase the infiltration of these inflammatory cells in general need to be explored as a future direction for successful treatment with CPI. As shown in other tumor entities, a dual blockade of CTLA-4 and PD-1 or PD-1/PDL-1 and LAG-3 could probably enhance the efficacy of CPI monotherapies, also by rescuing CD8+ T cells more vigorously from exhaustion than single signaling blockade (34, 35).

In case of contraindications for a CPI treatment and if oncogenic alterations are detected, targeted therapies should be discussed, although there is no experience with targeted therapies in PDS to date (2, 7, 8). In relation to this, rarely detected *PDGFRA* or *KIT* amplifications/mutations could be of interest as several drugs have proven to induce long-term remissions in PDGFR-expressing cancers, such as gastrointestinal stromal tumors,dermatofibrosarcoma protuberans, or myeloid malignancies (36–40). Furthermore, it has been shown that tumors with a loss of *CDKN2A/B* may benefit from CDK4/6 inhibitors, such as palbociclib, abemaciclib or ribociclib, all approved for the treatment of metastasized breast cancer (41–44).

In patients with advanced stage PDS treated with CPI, further investigation of predictors is still needed. However, all existing studies suggest a high efficacy of immune checkpoint blockade in inoperable or metastatic PDS patients.

## Author contributions

All authors contributed to the article and approved the submitted version.

## Funding

We acknowledge support by the Open Access Publication Fund of the University of Duisburg-Essen.

## Conflict of interest

The authors declare that the research was conducted in the absence of any commercial or financial relationships that could be construed as a potential conflict of interest.

The reviewer GL declared a shared affiliation with the author SK to the handling editor at the time of review.

## Publisher’s note

All claims expressed in this article are solely those of the authors and do not necessarily represent those of their affiliated organizations, or those of the publisher, the editors and the reviewers. Any product that may be evaluated in this article, or claim that may be made by its manufacturer, is not guaranteed or endorsed by the publisher.
